# The Role and Clinical Potentials of Circular RNAs in Prostate Cancer

**DOI:** 10.3389/fonc.2021.781414

**Published:** 2021-11-05

**Authors:** Mohammad Taheri, Sajad Najafi, Abbas Basiri, Bashdar Mahmud Hussen, Aria Baniahmad, Elena Jamali, Soudeh Ghafouri-Fard

**Affiliations:** ^1^ Skull Base Research Center, Loghman Hakim Hospital, Shahid Beheshti University of Medical Sciences, Tehran, Iran; ^2^ Student Research Committee, Department of Medical Biotechnology, School of Advanced Technologies in Medicine, Shahid Beheshti University of Medical Sciences, Tehran, Iran; ^3^ Urology and Nephrology Research Center, Shahid Beheshti University of Medical Sciences, Tehran, Iran; ^4^ Department of Pharmacognosy, College of Pharmacy, Hawler Medical University, Erbil, Iraq; ^5^ Institute of Human Genetics, Jena University Hospital, Jena, Germany; ^6^ Department of Pathology, Loghman Hakim Hospital, Shahid Beheshti University of Medical Sciences, Tehran, Iran; ^7^ Department of Medical Genetics, School of Medicine, Shahid Beheshti University of Medical Sciences, Tehran, Iran

**Keywords:** circular RNAs, prostate cancer, diagnosis, prognosis, biomarker

## Abstract

Globally, prostate cancer (PCa) is the second most commonly diagnosed cancer in men globally. Early diagnosis may help in promoting survival in the affected patients. Circular RNAs (circRNAs) are a novel class of non-coding RNAs (ncRNAs) which have been found to show extensive dysregulation in a handful of human diseases including cancers. Progressions in RNA identification techniques have provided a vast number of circRNAs exhibiting either up-regulation or down-regulation in PCa tissues compared to normal adjacent tissues. The mechanism of action is not clear for most of dysregulated circRNAs. Among them, function of a number of newly identified dysregulated circRNAs have been assessed in PCa cells. Increase in cell proliferation, migration, invasion, and metastasis have been reported for up-regulated circRNAs which suggest their role as oncogenes. On the other hand, down-regulated circRNAs have shown tumor suppressing actions in experimental studies. Furthermore, in a majority of studies, circRNAs have been found to sponge microRNAs (miRNAs), negatively regulating expression or activity of the downstream miRNAs. Additionally, they have been identified in interaction with regulatory proteins. This axis consequently regulates a signaling pathway, a tumor suppressor, or an oncogene. Easy, quick, and reliable detection of circRNAs in human body fluids also suggests their potentials as biomarker candidates for diagnosis and prediction of prognosis in PCa patients. In this review, we have discussed the role and potentials of a number of dysregulated circRNAs in PCa.

## Introduction

Prostate cancer (PCa) accounts for more than 1 in 5 new cancer cases in men (1). High age is the main risk factor for PCa. Race, environmental and genetic factors are known as other predisposing factors (2). Majority of PCa cases are diagnosed with diseases of low to intermediate risk, and a minority of 30% experience shorter survival in case of distant metastasis of the malignancy (3). Gleason score is the most commonly used grading system for prediction of outcomes in PCa patients (4). The scores range from 6-10 with high scores corresponding to malignant PCa cells and lower survival in PCa patients. The main biomarker currently used for diagnosis of PCa is prostate-specific antigen (PSA) which harbors good diagnostic values, however cannot distinguish PCa from resembling milder prostate disorders like benign prostate hyperplasia (BPH) and prostatitis (5). Non-coding RNAs (ncRNAs) have been studied in PCa and their role in development, progression, and metastasis of malignancy has been evaluated in a handful of *in vitro* and *in vivo* experiments. Among ncRNAs, microRNAs (miRNAs) and lncRNAs [see more at (6, 7)] have been studied more in PCa compared with circRNAs and their roles and mechanisms in development and progression of PCa have been clarified due to the historical priority of discovery and facility of detection in research studies.

## NcRNAs

A large proportion of eukaryotic genome encodes no protein which is termed as non-coding RNAs (ncRNAs). These transcripts have been primarily described as junk DNA and now known to have essential regulatory roles. Circular RNAs (circRNAs) are covalently closed RNA transcripts usually belonging to a novel type of ncRNAs, namely long non-coding category (lncRNAs). CircRNAs have been primarily reported in viroids (8). Hsu and Coca-Prados (9) reported the first circRNAs in eukaryotes in 1979 *via* electron microscopy in HeLa cells. Compared to linear types of regulatory RNAs and even among ncRNAs, less attention has been paid to circRNAs. However, increasing evidence demonstrates their biological functions. CircRNAs are believed to be formed *via* back-splicing from pre-messenger RNA (pre-mRNA) or originated from differentially spliced transcripts (10). The main characteristic of circRNAs as their names suggest is their determinant circular form which develops *via* covalent linkages between the 5′ and 3′ ends and sometimes 5′-2′ phosphodiester bonds. CircRNAs exhibit dissimilarities to conventional linear RNAs such as mRNAs and transfer RNAs (tRNAs) which include lacking capping and polyadenylated (poly A) tail at their 5′ and 3′ ends, respectively. Lack of open ends makes RNA loops resistant to RNA degrading enzyme RNase R which facilitates biochemical characterization of circRNAs (11). Several approaches based on non-polyadenylation characteristic of circRNAs, rRNA-depletion, and RNase R-resistance have been developed for detection of circRNAs (12). The qRT-PCR analysis following RNase R treatment is the most common approach used for validation of circRNAs (13). Also, fast and easy detection of circRNAs can be conducted through rolling-circle amplification (RCA) (14). This technique does not require any advanced equipment or fluorescent probes and is performed just using qRT-PCR machine and gel electrophoresis. First, the circRNAs using first-generation primers is reverse transcribed generating a multimeric cDNA through RCA method compared to a monomeric cDNA for linear RNA template. Then using second-generation primer, the circRNAs-specific ligation site is spanned and subsequently can be seen on gel electrophoresis (15). High-throughput RNA sequencing (RNA-seq) along with bioinformatics tools [e.g., CircMiner (16)] and confirmatory techniques like quantitative real-time polymerase chain reaction (qRT-PCR, where the junction/fusion site is known) and Fluorescence *in situ* hybridization (FISH) have helped substantial progression in identification of differentially expressed circRNAs in cancerous tissues. Although qRT-PCR is the most common approach for experimental detection of circRNAs, however, currently, no easy, quick, and cheap technique is available for diagnostics to detect a specific circRNA, and so it is suggested that we have a long way to bring circRNAs to clinical setting (17).

CircRNAs have been identified in large quantities and have been revealed to be expressed widely in animal cells. Thousands of circRNAs are transcribed in considerable fractions from a large number of human genes (18). The number of identified unique circRNAs are more than twice of the linear counterparts (19). However, they are usually found in lesser quantities compare with their linear counterparts (10). In mammals, circRNAs show conservation in their sequences among different species, are mainly found in cytoplasm, and demonstrate specific tissue, cellular and developmental stage distribution (20, 21), even more specific compared to corresponding mRNA isoforms (22). Precise functions of circRNAs have not been clarified, but regulatory roles have been described for an increasing number of them. The first circRNA, which its function has been characterized, was CDR1as (21). CDR1as was shown to play role in gene expression at posttranscriptional stage *via* binding to and sponging miR-7. This circRNA is involved in brain development (21). Aberrant expression of circRNAs have been associated with pathological conditions such as cardiovascular diseases (23), sudden cardiac death (SCD) (24), neurodegenerative and psychiatric disorders (25, 26), kidney diseases (27), inflammation (28), autoimmune diseases (29) and particularly various types of cancer. Cellular studies have shown a vast number of circRNAs to be dysregulated in cancer tissues compared to normal tissues and this imbalance can enhance tumor development and progression *via* affecting cell cycle. Aberrant expression includes either up- or down-regulation in levels of circRNAs compared to those in normal cells. Up-regulated circRNAs in cancer are known as oncogenes. These oncogenic circRNAs such as circMBOAT2 (30) and circFOXO3 (31) accelerate tumor cell proliferation, migration, invasion, and metastasis, while suppressing apoptosis. On the other hand, down-regulated circRNAs are considered as tumor suppressors. CircRNAs can be detected in high abundance due to their stability in body fluids like serum and urine, and can also be specifically expressed in extracellular vesicles (32, 33). Therefore, their detection provides easy, rapid, reliable, and minimally invasive diagnostic routes for many types of cancers and other pathological conditions. The expression levels of circRNAs in a majority of studies have been significantly correlated with clinicopathological features in cancerous patients, accordingly they can help in prediction of the disease prognosis. Most importantly, targeting oncogenic circRNAs or reversing intermediates using RNA interference (RNAi) and antisense oligonucleotides (ASO) or enhancing expression of circRNA with tumor suppressing functions has suggested new therapeutical potentials in fighting against malignancies. Thus, circRNAs have been introduced as novel diagnostic and prognostic biomarkers and therapeutical targets particularly in cancer.

In this review, we focus on a number of circRNAs being dysregulated in prostate cancer (PCa), with an overview of the studies assessed the tumorigenic and anti-carcinogenic roles of them in PCa tissues and finally, their potential applications in diagnosis and prediction of prognosis in PCa patients.

## CircRNAs in PCa

In various studies, thousands of circRNAs have been found to show aberrant expression in PCa tissues compared to normal adjacent tissue (NAT) or also between several stages of malignancy including primary and metastatic PCa (34, 35). Some of these circRNAs promote PCa cell tumorigenicity enhancing cell proliferation, invasion and metastasis. An increased resistance to chemotherapy agents is another impact of oncogenic circRNAs, which can affect the survival in PCa patients (36). Regulatory effects on expression of androgens or their receptors and resistance to them or reverse interactions which play role in development of PCa have also been reported for several circRNAs [e.g., circRNA-17 (37), circSMARCA5 (38), and circRNA-51217 (39)]. For some other circRNAs such as circ-ITCH (40) and circMBOAT2 (30), a correlation has been recognized between circRNA expression levels and prognosis in PCa patients. Precise mechanism of action for circRNAs has been poorly understood. In bioinformatics-constructed regulatory networks such as Gene Ontology (GO), Kyoto Encyclopedia of Genes and Genomes (KEGG) pathway, and competing endogenous RNA (ceRNA) network analyses to predict the function of circRNAs and also, RNA-pull down assays, an interaction between circRNAs and targeted miRNAs is frequently reported. Along with main miRNAs sponging activity, circRNAs also have been shown to exert their regulatory functions through interactions with proteins particularly RNA-binding proteins, which play role in RNAs maturation and influence various cellular functions (41). RNA-binding proteins can interplay with circRNA junctions and participate in splicing, processing, folding, stabilization, and localization of circRNAs (42). An example of circRNAs interacting with RNA-binding proteins are has_circ_0000020 (interacting with HuR, FMRP and EIF4A3) (43).

Also, interaction of circRNA with other ncRNAs particularly miRNAs, based on their complementary sequences has been reported in a set of experiments. Additionally, circRNAs can regulate RNA-binding proteins, and linear protein-coding mRNAs (44). These studies suggest an axis through which the circRNA affects (mainly reverses) expression or activity [see review in (45)] of a mediator miRNA as a target, which itself impacts a specific target signaling pathway, a tumor suppressor or an oncogene. Thus, it is thought that an oncogenic circRNA exerts its function through a signaling axis eventually activates an oncogene or through a signaling pathway accelerates cell cycle. Based on tissue, cancer or malignancy stage-specific expression of a circRNA in PCa, diagnostic analyses have unveiled reliability of a set of circRNAs as potential biomarkers in distinguishing PCa from any other condition or among cancer stages. Association between expression level of a circRNA and clinicopathological features in PCa patients like tumor stage, grade, lymph node metastasis in addition to prognosis, also has been found in a number of studies. For instance, Greene et al. (35) have reported that circRNAs not only demonstrate differential expression in PCa tissues, but also are expressed aberrantly according to the androgen dependency, which is known to play role in the pathogenesis of the disease. Furthermore, targeting aberrantly expressed circRNAs in PCa has exhibited hopeful results in a number of studies decreasing aggressiveness and tumorigenesis in cell and *in vivo* studies.

## Up-Regulated circRNAs in PCa

Up-regulated circRNAs, as discussed above, act as oncogenic ncRNAs promoting tumorigenic features of tumor cell lines and also, increase tumor progression *in vivo*. CircRNA knockdown using specific small interfering RNA (siRNA) or overexpression by encoding vectors have been employed in functional analysis. Enhanced tumor cell proliferation, invasion and migration have been reported following over-expression of these circRNAs, in cell viability and colony formation, migration and invasion assays, respectively. Suppressed apoptosis is also reported in functional analysis of up-regulated circRNAs. Furthermore, oncogenic circRNAs could decrease chemosensitivity and radiosensitivity of the cancer cell lines to the current therapeutical approaches of PCa (46, 47). As a result, decreased effectiveness of the cancer therapeutics and eventually adverse consequences such as shortened survival is predicted for the patients.

For instance, Zhang et al. (48) have identified 89 circRNAs which showed aberrant expression in PCa tissues, among them 32 circRNAs showed increased expression and remaining 57 demonstrated to be down-regulated. In further investigations, 5 prominently overexpressed circRNAs in comparison to their corresponding mRNAs in PCa tissues including hsa_circ_0006754, hsa_circ_0005848, hsa_circ_0006410, hsa_circ_0003970, and hsa_circ_AKAP7 were recognized. Interaction networks revealed 215 linkages between 5 selected circRNAs and corresponding miRNAs. Several miRNAs including miR-204-5p, miR-3160-5p, and miR-548 were identified as the most prominent targets of associated circRNAs which play role in suppression or promotion of carcinogenesis or *via* enhancing apoptosis, inhibiting cell proliferation or PI3K/AKT signaling pathway, respectively. MAPK signaling pathway was known as the most important signaling pathway affected by the selected circRNAs, while other pathways like hormone-associated and lipid metabolism-related were also involved in the carcinogenic axes of the highly expressed circRNAs. Survival rate analysis in PCa patients by the Kaplan-Meier curve unveiled a positive correlation between higher expression of cognate genes corresponding to anti-carcinogenic circRNAs (hsa_circ_0006410, hsa_circ_AKAP7 and hsa_circ_0005848) and better overall survival (OS) in PCa patients. Also, Yu et al. (49) identified 13 circRNAs in association with resistance to enzalutamide as an androgen deprivation therapy (ADP) drug used against PCa. Six miRNAs, 167 mRNAs, and 10 hub genes were identified as targets of the circRNAs. Among them, 8 prognostic-associated mRNAs were shown to be associated with survival rates in PCa patients, also with an AUC of 0.816 confirming the accuracy of miRNAs signature in detection of PCa. Additionally, knockdown experiments revealed that circRNA hsa_circ_0047641 promote PCa cell proliferation, migration, and invasion. CircMBOAT2 is another circRNA participating in the pathogenesis of PCa through increasing cell proliferation, migration, and invasion of malignant cells. This circRNA significantly up-regulates mTOR expression through sequestering miR-1271-5p, leading to the activation of the PI3K/Akt cascade (50).

CircSLC19A1 is another up-regulated circRNA in PCa tissues. CircSLC19A1 knock down has suppressed viability of PCa cells and their proliferation through modulation of miR-326/MAPK1 axis (51). CircABCC4 is an example of up-regulated circRNAs in PCa tissues and cell lines which enhances expression of FOXP4 through sequestering miR-1182. CircABCC4 silencing has inhibited proliferation of PCa as well as their migratory potential and invasiveness. Besides, circABCC4 knock down has attenuated growth of PCa *in vivo*. Cumulatively, circABCC4 accelerates malignant behavior of PCa (52).

**Table 1** summarizes the studies on a set of up-regulated circRNAs in PCa.

**Table 1:** Up-regulated circRNAs in PCa.

circRNA (Other terms)	Clinical Cases	Cell Lines	Target genes/Regulators/sponged miRNAs	Affected Signaling Pathway/Process	Findings on over-expressed or silenced circRNA in PCa cellular experiments	Ref. (s)
hsa_circ_0006754, hsa_circ_0005848, hsa_circ_0006410, hsa_circ_0003970, and hsa_circ_AKAP7 signature	2 PCa patient tissues and matched NATs for RNA-Seq and also, 20 PCa patients and matched NATs for qRT-PCR verification	–	miR-204-5p, miR-3160-5p, and miR-548	PI3K-AKT, MAPK, hormone and lipid-related pathways	–	(29)
CircABCC4	47 PCa tissues and paired NATs	PC3 and DU145 human PCa cell lines	miR-1182/FOXP4 signaling axis	–	Its silencing suppressed PCa cell proliferation, migration and invasion *in vitro* and tumor propagation *in vivo*	(53)
circMBOAT2 (has_circ_0007334)	Two cohorts (cohort1: 50 PCa patient tissues and paired NATs; cohort 2: 62 PCa patients)	PC-3, DU145, VCaP, LNCaP, and C4-2B PCa cell lines and RWPE-1 healthy prostate epithelial cells	miR-1271-5p/mTOR axis	mTOR	circMBOAT2 depletion using specific siRNA inhibited PCa cell proliferation, migration and invasion *in vitro* and overexpression showed reverse effects and also, tumor progression and metastasis *in vivo*	(30)
circFOXO3	53 PCa tissues and paired NATs	LNCaP, 22Rv1, DU145, PC-3 and WPMY‐1	miR-29a‐3p/SLC25A15 cascade		circFOXO3 knockdown suppressed PCa cell proliferation and increased apoptosis though making arrest in cell cycle	(31)
circ_0057558	25 PCa tissues	22RV1, DU145, and PC3 human PCa cell lines and 293T human kidney epithelial cells	miR-206/USP33/c-Myc axis	–	Circ_0057558 silencing inhibited PCa cell proliferation and colony formation, and caused arrest in cell cycle *in vitro* and tumor growth *in vivo* Overexpression attenuated sensitivity to chemotherapy agent docetaxel	(46)
circ_0088233	46 PCa tissues and paired NATs	22Rv1, Du145, LNCaP, and RWPE-1	miR-185-3p	–	circ_0088233 knockdown inhibited PCa cell proliferation, migration and invasion and induced G1 phase cell cycle arrest and also, apoptosis	(54)
circMYLK	17 PCa tissues and paired NATs	DU145, LNCaP, PC-3, PC-3MIE8 and RWPE-1	miR-29a	–	circMYLK up-regulation promoted PCa cell proliferation, migration and invasion and suppressed apoptosis	(55)
circHIPK3 (hsa_circ_0000284)	26 PCa tissues and paired NATs	LNCaP, PC3, DU145, 22Rv1, and RWPE-1	miR-193a-3p/MCL1 axis	–	circHIPK3 knockdown inhibited PCa cell proliferation and invasion *in vitro* and tumor growth *in vivo*	(56)
	60 PCa tissues and paired NATs	22RV1, PC-3, DU145, LNCaP, and RWPE-1	miR-338-3p/ADAM17 axis	–	circHIPK3 knockdown inhibited PCa cell proliferation and invasion	(57)
	Serum samples of 35 PCa tissues and 35 heathy volunteers	RWPE-1, 22Rv1, and DU145	miR-212/BMI-1 axis	–	circHIPK3 knockdown suppressed PCa cell proliferation, migration and invasion and enhanced apoptosis *in vitro* and tumor growth *in vivo*	(58)
circ0005276	90 PCa tissues and paired NATs	PC-3, DU145, VCaP, LNCaP, and RWPE-1 cells	FUS/XIAP axis	–	circ0005276 knockdown inhibited PCa cell proliferation and migration	(59)
circ_0006404	30 PCa tissues and paired NATs	LNCaP-AI and DU145 LNCaP, and WPMY-1	miR-1299/CFL2 axis	–	circ_0006404 knockdown suppressed PCa cell proliferation, viability and metastasis and increased apoptosis *in vitro* and also, inhibited tumor growth *in vivo*	(60)
circ-ZNF609	30 PCa tissues and paired NATs	22Rv1, LNCaP, DU145, VCaP and RWPE-1	miR-501-3p/HK2 axis	–	circ-ZNF609 knockdown supressed tumor progression and radioresistance in PCa cell lines *in vitro* and also, increased radiosensitivity *in vivo*	(47)
circ-SMARCA5	21 PCa tissues and paired NATs	22RV1, DU145, PC-3, LNCaP and WPMY-1	–	–	circ-SMARCA5 knockdown inhibited PCa cell proliferation, and induced apoptosis *via* G1 phase cell cycle arrest	(38)
circ-102004	16 PCa tissues and 6 BPH tissues	PC3 and 22RV1 PC3 and 22RV1	–	ERK, JNK, Hedgehog, AKT, and Wnt/β-Catenin	Overexpression increased PCa cell proliferation, migration, and invasion and inhibited apoptosis *in vitro*, also increased tumor progression *in vivo*	(61)
circPDHX (hsa_circ_0003768)	75 PCa tissues and paired NATs	PC3 and 22RV1 PC3 and 22RV1	miR-378a-3p/IGF1R axis	–	circPDHX knockdown inhibited PCa cell proliferation, colony formation and invasion *in vitro* and tumor progression *in vivo*	(62)
circAGO2	24 PCa tissues and paired NATs	PC-3	HuR	–	circAGO2 overexpression increased PC-3 cell proliferation and invasion *in vitro* and tumor growth *in vivo*	(59)
circ-TRPS1	80 PCa tissues and paired NATs	PC3, LnCaP, DU145 and RWPE-1	miR-124-3p/EZH2 axis	–	circ-TRPS1 knockdown inhibited PCa cell proliferation and invasion *in vitro* and *in vivo*	(63)
circNOLC1	80 PCa tissues and 16 NATs	DU145, PC3, C4-2, LNCaP, 22RV1, and RWPE1	miR-647/PAQR4 axis	As downstream of NF-kB	circNOLC1 overexpression increased PCa cell proliferation and migration *in vitro* and knockdown reversed the effects, also increased tumor growth *in vivo*	(62)
circ_0057553	37 PCa tissues and paired NATs	22RV1, PC3, DU145, LNCap, and RWPE1	miR-515-5p/YES1 axis	Glycolysis	circ_0057553 silencing suppressed PCa cell proliferation, migration, and invasion, also increased apoptosis *in vitro.* Furthermore, inhibited tumor progression *in vivo*	(64)
circ-0016068	42 PCa tissues and paired NATs	DU 145, 22RV1, PC-3, VCaP, and RWPE1	miR-330-3p/BMI-1 axis	–	circ-0016068 knockdown suppressed PCa cell proliferation, migration, invasion, and EMT *in vitro*, also inhibited tumor growth and metastasis *in vivo*	(65)
circ_CCNB2	25 PCa tissues and paired NATs	DU145 and LNCaP	miR-30b-5p/KIF18A axis	–	circ_CCNB2 knockdown improved PCa cell radiosensitivity by suppressing autophagy *in vitro* and *in vivo*	(66)
circ_0062020	60 PCa tissues and 30 NATs	DU145, LNCaP, and WPMY-1	miR-615-5p/TRIP13 axis	–	circ_0062020 knockdown induced radiosensitivity in PCa *in vitro* and also, inhibited tumor growth *in vivo*	(16)
circSLC19A1	48 PCa tissues and paired NATs	DU145 cells, PC3 cells, LNCaP cells, 22RV1, and RWPE-1	miR-326/MAPK1 axis	–	circSLC19A1 knockdown suppressed PCa cell viability, proliferation, migration and invasion	(51)
	–	22Rv1, DU145, LNCaP, PC3, and WPMY-1	miR-497/SEPT2/ERK1/2 axis	ERK1/2	circSLC19A1 overexpression promoted PCa cell proliferation and invasion	(33)
hsa_circ_0000735	50 PCa tissues and paired NATs	PC-3, DU145, and RWPE-1	miR-7	–	hsa_circ_0000735 knockdown increased sensitivity to docetaxel in resistant PCa cells, also *in vivo*	(67)
circFMN2	90 PCa tissues and paired NATs	PC-3, DU145, VCaP, LNCaP, and RWPE-1	miR-1238/LHX2 axis	–	circFMN2 knockdown decreased PCa cell proliferation, colony formation, migration and invasion *in vitro* and also, tumor growth *in vivo*. circFMN2 exerts its hyperproliferative role *via* increasing DNA synthesis and suppressing apoptosis.	(32)
circPVT1	43 PCa tissues and 15 paired NATs	DU145	MYC oncoprotein	–	circPVT1 knockdown inhibits *MYC* expression in PCa cells. circPVT1 stabilizes the MYC protein in high grade of Gleason score (GP4)	(68)
circRNA-51217	–	PC3, LNCaP, C4-2, DU145, and HEK293T	miR-646	TGFβ1/p-Smad2/3	circRNA-51217 promoted PCa cell invasion	(39)
circZMIZ1	Serum samples of 14 PCa patients and paired NATs	DU145, C4-2, LNCaP, 22RV1, and RWPE-1	AR-V7	–	circZMIZ1 knockdown suppressed PCa cell proliferation by making arrest in G1/S phase of cell cycle. circZMIZ1 expression increased in response to androgens.	(69)
circGOLPH3	–	PC-3, and RWPE-1	CBX7	–	circGOLPH3 overexpression promoted PCa cell proliferation and suppressed apoptosis	(70)
circRNA_100146	–	WPMY1, DU145, LNCaP, 22RV1, VCaP, and PC-3	miR-615-5p/TRIP13 axis		circRNA_100146 knockdown suppressed PCa cell proliferation and metastasis *in vitro* and tumor growth *in vivo*	(71)
circXPO1	48 PCa tissues and 15 paired NATs	WPMY-1, PC-3, DU145, and 22RV	miR-23a	–	circXPO1 overexpression promoted PCa cell proliferation, colony formation, and invasion	(72)
circGNG4	40 PCa tissues and 15 paired NATs	RWPE, PC-3, LNCaP, VCaP, and DUL145	miR-223/EYA3/c-Myc axis	–	circGNG4 knockdown inhibited tumor cell proliferation, clonal formation, migration, and invasion *in vitro* and tumor growth *in vivo*	(73)
circ-XIAP	52 PCa tissues	RWPE, 22Rv1, VCaP, DU145, and PC3	miR-1182/TPD52 Axis	–	circ-XIAP knockdown increased sensitivity to Docetaxel in the drug-resistant PCa cells. circ-XIAP knockdown suppressed PCa growth and improved drug sensitivity *in vivo.*	(74)
circDPP4	104 PCa tissues and matched NATs	RWPE, PC3, DU145, LNCaP and 22RV1	miR-195/cyclin D1 axis	–	circDPP4 knockdown suppressed PCa cell proliferation, migration, and invasion *in vitro* and tumor growth *in vivo*	(75)
hsa_circ_0047641	–	REPW-1, PC3, LNcap, and DU145	–	–	hsa_circ_0047641 knockdown suppressed proliferation, migration, and invasion in enzalutamide-resistant PCa cells	(49)
circ_0001686	30 PCa tissues and matched NATs	REPW-1, CWR22RV1 and LNCaP	miR-411-5p/SMAD3/TGFBR2 axis	TGFβ	Overexpression of sponged miR-411-5p suppressed PCa cell proliferation, migration, and invasion *in vitro*, also inhibited tumor growth and metastasis *in vivo*	(76)

## Down-Regulated circRNAs in PCa

Decreased expression in high throughput RNA analyses of PCa tissues compared to paired NATs draws attention to a second group of dysregulated circRNAs. Down-regulated circRNAs exhibit anti-oncogenic behaviors in experimental analyses inhibiting proliferation, migration, invasion, and metastasis of PCa cells. Sponge activity is seen for a majority of circRNAs which mainly show reverse regulation on downstream miRNAs. Inhibited miRNAs are mainly oncogenic RNAs which activate their corresponding oncogenes or inactivate related tumor suppressors. Mediator miRNAs, predominantly act *via* a signaling axis which firstly regulate expression a downstream protein, and itself makes changes to a signaling pathway. Affected known signaling pathways in PCa like MEK/ERK and Wnt/β-Catenin have been reported in a handful of studies on down-regulated circRNAs (see **Table 2**).

**Table 2:** Down-regulated circRNAs in PCa.

circRNA (Other terms)	Clinical Cases	Cell Lines	Target genes/Regulators/sponged miRNAs	Affected Signaling Pathway/Process	Findings on over-expressed or silenced circRNA in PCa cellular experiments	Ref. (s)
circ-ITCH	52 PCa tissues and paired NATs	C4-2, LNCaP, DU145, 22Rv1, VCaP, and RWPE-1	miR-17-5p/HOXB13 axis	–	circ-ITCH overexpression inhibited PCa cell proliferation and increased apoptosis *in vitro*, also repressed tumor growth *in vivo*.	(77)
	–	VCaP, DU 145, PC-3, 22RV1, and RWPE-1	miR-197	–	circ-ITCH overexpression inhibited cell proliferation and increased apoptosis in PC-3 cells.	(78)
	10 PCa tissues and paired NATs	PC3, LNCaP, and RWPE-1	miR-17	Wnt/β-Catenin and PI3K/AKT/mTOR	circ-ITCH overexpression suppressed PCa cell viability and invasion.	(79)
hsa_circ_0001206	50 PCa tissues and paired NATs	PC-3, DU145, and LNCaP, and RWPE-1	miR-1285-5p	–	hsa_circ_0001206 overexpression suppressed PCa cell proliferation, colony formation, migration, and invasion *in vitro* and also, tumor growth *in vivo*.	(80)
circAMOTL1L (has_circRNA_000350)	3 PCa patient tissues including 35 BPH tissues, 34 low-grade PCa tissues and 28 high-grade PCa tissues	PC3, LNCaP, 1 DU145, and RWPE-1	miR-193a-5p/protocadherin-α axis	–	circAMOTL1L knockdown promoted PCa cell migration and invasion *in vitro* and conversely, overexpression decreased tumor growth *in vivo*. circAMOTL1L regulates EMT-related genes.	(81)
circUCK2	–	C4-2	miR-767-5p/TET1 axis	–	circUCK2 overexpression inhibited PCa cell proliferation, and invasion *in vitro* and tumor growth *in vivo*.	(82)
circ_LARP4	55 PCa tissues and paired NATs	LNCaP, DU145, and 22Rv1	–	–	circ_LARP4 up-regulation suppressed PCa cell migration and invasion, also induced expression of tumor suppressor FOXO3.	(83)
circ-MTO1 (has_circ_0076979)	298 PCa tissues and paired NATs	DU-145, VCaP, PC‐3, and RWPE-1	miR‐630 and miR‐17‐5p	–	circ-MTO1 overexpression inhibited PCa cell proliferation and invasion.	(84)
circ_KATNAL1 (hsa_circ_0008068)	–	LNCaP, DU145, and PC3, and WPMY-1	miR-145-3p/*WISP1* axis	–	circ_KATNAL1 overexpression suppressed PCa cell proliferation and invasion, and also, induced apoptosis. circ_KATNAL1 plays its role *via* regulatory effects on expression of caspases and matrix metalloproteases (MMPs).	(85)
circRNA17 (hsa_circ_0001427)	–	CWR22Rv1, C4–2, and 293T	miR-181c-5p/ARv7 axis	–	circRNA17 reversely regulates expression of androgen receptor variant-7 and accordingly, negatively affects PCa cells invasion and resistance to enzalutamide. circRNA17 overexpression inhibited tumor growth and metastasis *in vivo.*	(37)
circPSMC3	55 PCa tissues and paired NATs	DU145, PC3, LNCap and P69	DGCR8	–	circPSMC3 overexpression repressed PCa cell proliferation through negative regulation of cell cycle.	(86)
circDDX17	20 PCa tissues and paired NATs	22Rv1 and PC-3	miR-346/LHPP axis	–	circDDX17 overexpression inhibited PCa cell proliferation, migration, and EMT.	(85)
circSLC8A1 (hsa_circ_0000994)	15 PCa tissues and paired NATs	DU145, 22Rv1, LNCaP, PC-3 and WPMY-1	miR-21	MAPK and chemokine pathways	circSLC8A1 knockdown increased PCa cell proliferation and migration.	(87)
circCRKL (hsa_circ_0001206)	45 PCa tissues and paired NATs	DU145, C4- 2, 22Rv1, LNCaP, and RWPE-1	miR-141/KLF5 axis	–	circCRKL overexpression inhibited PCa cell migration and invasion *via* suppressing cell cycle and increased apoptosis *in vitro* and also, inhibited tumor growth *in vivo*.	(88)
circ_17720 and circ_14736	144 PCa tissues and paired NATs	PC3, DU145, C4-2, and LNCaP, and RWPE-1	–	–		(89)
hsa_circ_0075542	30 PCa tissues and paired NATs	LNCaP and PC3	miR-1197/HOXC11 axis	–	hsa_circ_0075542 overexpression inhibited cell proliferation, migration, and invasion and enhanced apoptosis	(90)
circSMARCA5	20 PCa tissues and paired NATs	RWPE-1, DU145, LNCaP, and PC3	miR-181b-5p + miR-17-3p/TIMP3 axis	–	circSMARCA5 overexpression inhibited PCa cell proliferation, migration, and invasion *in vitro* and suppressed tumor growth and metastasis *in vivo*.	(91)
circSLC8A1 (hsa_circ_0000994)	15 PCa tissues and paired NATs	WPMY-1, PC-3, 22Rv1, DU145, and LNCaP	miR-21	–	circSLC8A1 knockdown promoted PCa cell proliferation and migration	(87)

Circular RNA Itchy E3 ubiquitin protein ligase (circ-ITCH) is an example for down-regulated circRNAs in PCa which has been studied in four distinct experiments (77–79). Wang et al. (92) demonstrated that circ-ITCH down-regulation increases PCa cell proliferation and decreases apoptosis *in vitro*, while its up-regulation decreases cell proliferation and *in vivo* tumor growth. Luciferase assay showed direct interaction of circ-ITCH with microRNA miR-17-5p and reverse relationship between their expression levels, which reveals that circ-ITCH acts as sponge for downstream miR-17-5p. miR-17-5p, itself negatively regulates expression of HOXB13, which is known as a tumor suppressor gene being involved in development and progression of PCa. Yuan et al. (78) showed the same experimental results about consequences of circ-ITCH down-regulation, although miR-197 was identified as target miRNA for circ-ITCH. Also, miR-17 was detected as target of circ-ITCH in a study by Li et al. (79). Furthermore, they demonstrated that down-regulation of circ-ITCH is associated with up-regulation of expression of proteins involved in β-catenin, p-AKT, and p-mTOR signaling pathways indicating that circ-ITCH negatively regulates these pathways which have role in the progression of various tumors like PCa. In another study, Huang et al. (40) assessed the correlation between circ-ITCH expression and clinicopathological features, survival and prognosis in PCa. Direct association between low circ-ITCH levels and more aggressive clinicopathological features, poor survival, and unfavorable prognosis confirmed the experimental studies identifying circ-ITCH as a tumor suppressor circRNA in PCa. circAMOTL1L is another down-regulated circRNA in PCa. Its down-regulation has promoted PCa cell migration and invasion *in vitro*, while its overexpression has decreased tumor growth *in vivo*. circAMOTL1L has been shown to regulate expression of EMT-related genes (81).

**Figure 1:**
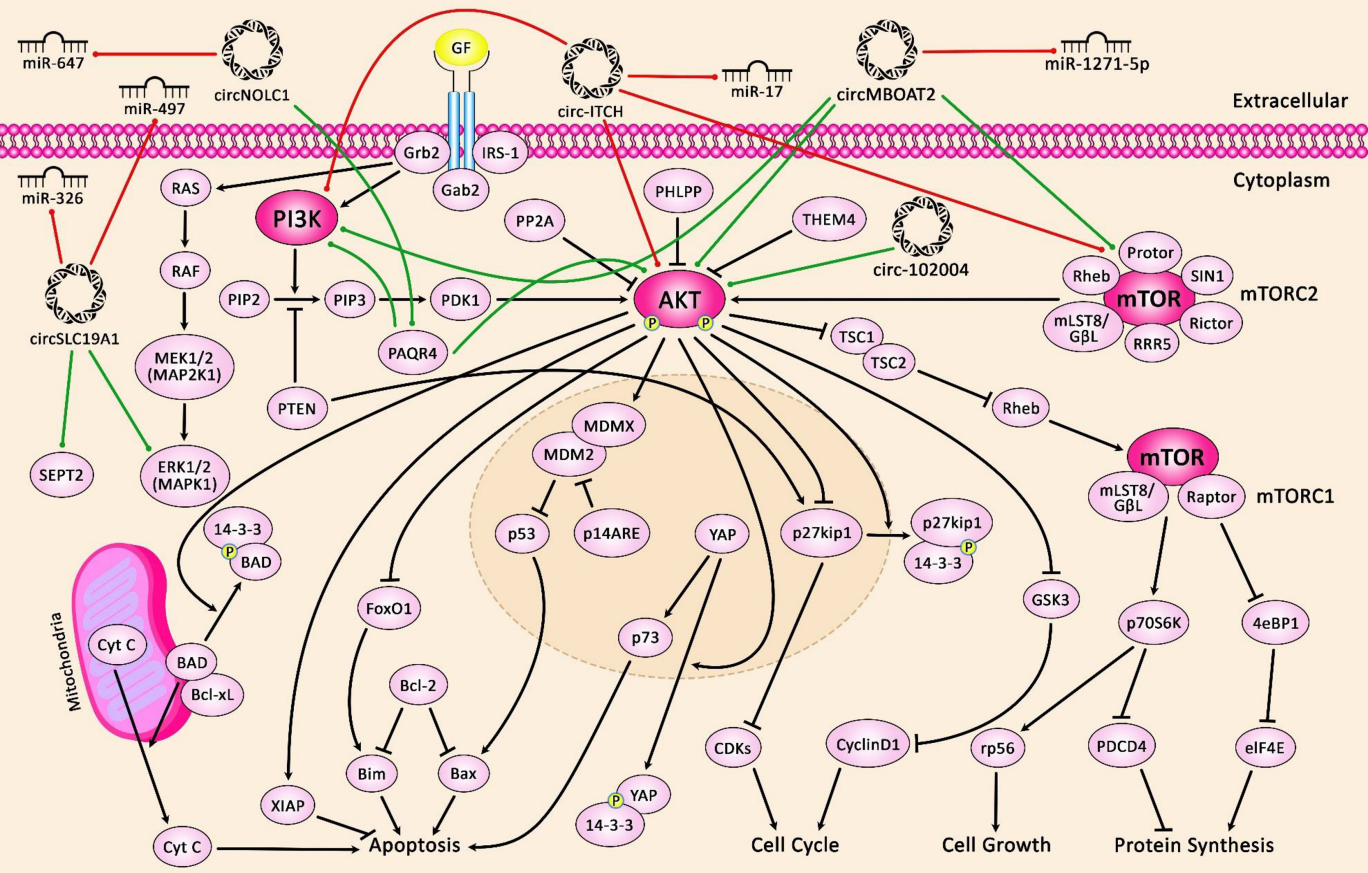
A schematic diagram of the crosstalk between circRNAs and PI3K/AKT/mTOR and MAPK/ERK signaling pathways in prostate cancer. The figure suggests that extracellular circRNAs enter cells.

**Figure 1** illustrates the role of several circRNAs in PCa *via* modulating the PI3K/AKT/mTOR and MAPK/ERK pathways.

PI3K signaling cascade linking RTK signaling results in downstream activation of PI3K/AKT/mTOR, elevating cell proliferation and survival. Besides, the MAPK-ERK signaling cascade also called the Raf/MEK/ERK pathway, is the main signal pathway of the MAPK signal cascade. The main MAPK/ERK kinase kinase (MEKK) components are the Raf family members Raf-1, A-Raf and B-Raf. Activated Raf activates MEK-1/2 by phosphorylating serine residues. Moreover, MEK-1/2 upregulates ERK-1/2 through phosphorylating the threonine and tyrosine residues of ERK-1/2. Activated ERK can regulate the phosphorylation of some nuclear transcription factors that are directly involved in the modulation of cell proliferation and differentiation. A recent study has demonstrated that overexpression of circMBOAT2 significantly upregulates mTOR expression *via* sponging miR-1271-5p, leading to the activation of the PI3K/Akt cascade, eventually elevating the cell proliferation, migration, and invasion of prostate cancer (50). Another research has denoted that circSLC19A1 elevates the expression level of MAPK1 by downregulating the miR-326 expression, thereby promoting prostate cancer cell proliferation, migration and invasion (51). Green arrows indicate upregulation of target genes modulated *via* circRNAs; red arrows depict inhibition by them.

## Diagnostic and Prognostic Applications of circRNAs in PCa

As discussed above, circRNAs are found in extracellular vesicles extracted from plasma, exhibit high resistance to degradation and so, are detected in high concentration in easily received liquid biopsies suggesting potential application as biomarkers in diagnosis of various types of cancer (93). Early, quick, and minimal or non-invasive diagnosis based on detection of dysregulated circRNAs in human bio-specimens like blood and urine (94) makes it possible to provide scheduled and real time monitoring of the responses to treatment and prediction of prognosis in PCa patients. Accordingly, early treatment improves patient survival and so, good prognosis could be predicted for the patients. Both classes of dysregulated circRNAs including up- and down-regulated types can be detected in PCa patient samples and used as clinical biomarkers.

Wang et al. (89) have developed the first bioinformatics-based prognosis model for prediction of biochemical recurrence (BCR) in PCa which used a signature comprised of 8 circRNAs. Among them, two circRNAs including circ_17720 and circ_14736 were detected in exosomes extracted from plasma samples of PCa patients. Furthermore, both exhibited down-regulation in PCa tissues compared to NATs. Experimental analyses revealed that they repress PCa cell proliferation. Survival analysis *via* Kaplan-Meier for eight circRNAs showed that up-regulated circRNA correlated with unfavorable BCR-free survival and those with down-regulation changes were associated with less BCR. The area under curve (AUC) in Receiver-operating characteristic (ROC) for the signature was reported to be 0.799.

Greene et al. (95) profiled circRNAs in enzalutamide-chemoresistance in LNCaP PCa cell lines using high-throughput RNA sequencing. In bioinformatics analyses, five aberrantly expressed circRNAs were identified in enzalutamide-resistant LNCaP cells. Among them, hsa_circ_0004870 showed diminished expression in cells with high levels of androgen receptor (AR) compared to low AR-expressing cells and also, in malignant cells related to benign LNCaP cells. The corresponding *BMP39* gene was also showed down-regulation in enzalutamide-resistant cells. Chen et al. (96) showed that a circRNA signature not only can distinguish PCa tissues from healthy prostate tissues, but also help distinguishment of PCa subtypes. Several circRNAs have shown dysregulation in accordance with Gleason score or correlated with advancement of clinicopathological features in PCa patients (see **Table 3**). Mao et al. (98) demonstrated that increased circPDHX expression levels in PCa tissues is correlated with malignant clinicopathological features in PCa patients. Kaplan-Meier analysis to assess the association between circPDHX expression and prognosis revealed that PCa patients with elevated circPDHX levels had poorer survival relative to patients with low levels. Univariate and multivariate regression analyses also showed that circPDHX high expression level along with advanced Gleason score act as independent prognostic factors for PCa patients predicting poorer survival. In diagnostic analyses, acceptable values of AOC in ROC curve, sensitivity, and specificity of 0.64, 80.0%, and 58.7%, respectively, were found for circPDHX showing promising results especially in distinguishment of PCa from healthy people. He et al. (100) evaluated expression of circrNAs in urinary extracellular vesicles. Their study has indicated the accuracy of a urine circRNA classifier (Ccirc) composed of circPLXDC2, circSCAF8, circPDLIM5, circCCNT2, and circSCAMP1 in detection of PCa. Their results demonstrated higher accuracy of Ccirc compared to that for two care risk calculators. Also, the Ccirc showed better value in prediction of high grades of PCa in combination with risk calculators relative to that of standards alone. In another study, Zhong et al. (97) identified 160 autophagy-related circRNAs, then constructed a circRNA signature containing five circRNAs hsa_circ_0001747, hsa_circ_0002100, hsa_circ_0000280, hsa_circ_0000437, and hsa_circ_0001085 with aberrant expression between high risk and low risk groups of PCa patients. Univariate and multivariate Cox regression analyses demonstrated the signature as an independent prognostic indicator in PCa patients. Also, ROC curve showed higher AUC values for the signature compared to conventional indicators like PSA, age, clinicopathological T stage, and Gleason score. Among the signature circRNAs, hsa_circ_0001747 was identified in association with a higher number of autophagy-related genes and its knockdown in experimental validation promoted PCa cell proliferation *in vitro* and *in vivo* through autophagy augmentation.

**Table 3:** circRNAs with diagnostic or prognostic values in PCa.

Description	Area under Curve (AOC)	Sensitivity	Specificity	Kaplan–Meier analysis	Univariate Cox regression	Multivariate cox regression	Other correlation tests	Ref. (s)
5-circRNA signature	–	–	–	Higher expression of cognate genes for hsa_circ_0006410, hsa_circ_AKAP7 and hsa_circ_0005848 positively correlated with better OS in PCa patients.	–	–	–	(29)
5-circRNA signature	0.827	–	–	PCa patients at the high-risk group with higher expression of hsa_circ_0000437, hsa_circ_0000280, and circ_5017 had lower survival compared to the low-risk group.	Autophagy-related prognostic risk score was correlated with survival in PCa patients.		–	(97)
circABCC4 expression in PCa patients	–	–	–	Higher circABCC4 levels positively correlated with survival in PCa patients	–	–	The Chi-square test showed association between circABCC4 expression and advanced clinicopathological features including higher tumor stage and metastasis	(53)
circMBOAT2 expression in a cohort including 50 PCa patients and matched NATs (high: 31 samples, low: 31 samples)	–	–	–	High circMBOAT2 expression correlated with shorter DFS in two cohorts of PCa patients	Increased expression significantly associated with poorer prognosis in PCa patients.		The *χ* ^2^ test demonstrated positive correlation between circMBOAT2 expression and advanced clinicopathological features including higher pathological T stage and Gleason score in PCa patients	(30)
circFOXO3 *via* SLC25A15 axis	–	–	–	High SLC25A15 expression correlated with shorter OS in PCa patients	–	–	- High SLC25A15 circFOXO3 expression was significantly associated with advanced Gleason score in PCa patients - Increased expression of SLC25A15 correlated with poorer prognosis	(31)
circ_0057558	–	–	–	circ_0057558 knockdown in increased OS nude mice	–	–	–	(46)
circHIPK3	–	–	–	–	–	–	High circHIPK3 expression predicted poorer prognosis for PCa patients	(56)
	–	–	–	–	–	–	miR-338-3p high expression levels as target of circHIPK3 correlated with histological grade, lymph node metastasis and distant metastasis.	(57)
circPDHX	0.64	80.0%	58.7%	PCa patients with elevated circPDHX expression levels had poorer survival relative to patients with low levels	circPDHX high expression level along with advanced Gleason score act as independent prognostic factors for PCa patients predicting poorer survival.		High circPDHX expression significantly correlated with high pathological T stage and Gleason score in PCa patients	(98)
circ-TRPS1	–	–	–	–	–	–	The Pearson’s correlation test showed an association between increased circ-TRPS1 expression and advanced clinicopathological features including higher T stage, lymph and distant metastasis. High circ-TRPS1 expression correlated with poorer prognosis in PCa patients	(63)
circ-0016068	–	–	–	PCa patients with elevated circ-0016068 expression exhibited decreased survival compared to those with lower levels.	–	–	The Fisher’s exact test showed correlation between high circ-0016068 expression and advanced clinicopathological features including higher pathological T stage, Gleason score, and lymph node metastasis.	(65)
circ_0062020	–	–	–	–	–	–	The Chi-square test showed correlation between high circ_0062020 expression and advanced clinicopathological features including higher tumor size, TNM stage, Gleason score and lymph node metastasis in PCa patients.	(99)
hsa_circ_0000735	–	–	–	Patients with high hsa_circ_0000735 expression showed shorter OS compared to those with low levels.	–	–	–	(67)
circFMN2	–	–	–	–	–	–	High circFMN2 expression showed association with high pathological T stage, lymph node and distant metastasis. circFMN2 was detected in exosomes extracted from serum of PCa patients.	(32)
circ-ITCH	–	–	–	Low circ-ITCH level significantly correlated with poorer OS in PCa patients.	–	–	Low circ-ITCH expression correlated with advanced clinicopathological characteristics including higher tumor stage, Gleason score and high PSA levels.	(77)
	0.812	88.3%	61.7%	PCa patients with high circ-ITCH expression had better survival including longer DFS and also improved OS.	High circ-ITCH levels were associated with longer DFS in PCa patients.	circ-ITCH level acts as an independent prognostic factor.	Low circ-ITCH levels correlated with unfavorable clinicopathological features including advanced pathological T stage and lymph node metastasis.	(78)
hsa_circ_0001633, hsa_circ_0001206, and hsa_circ_0009061	0.809, 0.774, and 0.711, respectively	–	–	–	–	–	hsa_circ_0001206 and hsa_circ_0009061 expression levels correlated with high Gleason score and tumor stage in PCa patients.	(80)
circ_LARP4	–	–	–	PCa patients with decreased circ_LARP4 expression levels exhibited poorer OS and worse prognosis compared to those with high levels.	–	–	–	(83)
circ-MTO1	–	–	–	PCa patients with high circ-MTO1 levels showed longer OS and DFS.	High circ-MTO1 levels predicted favorable OS and prognosis.	circ-MTO1 expression act as an independent predictive factor predicting better prognosis.	High circ-MTO1 expression levels were significantly associated with better clinicopathological features including lower pathological T stage and N grade in PCa patients.	(84)
circPSMC3	–	–	–	PCa patients with low circPSMC3 expression levels exhibited worse prognosis compared to those with high levels.	–	–	–	(86)
circSLC8A1	–	–	–	–	–	–	Patients with low circSLC8A1 expression exhibited shorter survival compared to those with high levels.	(87)
circRNA signature	0.799	–	–	Up-regulated circRNAs in the signature were correlated with less survival and down-regulated circRNAs were significantly associated with less recurrence.	–	–	–	(89)

The prognostic value of circRNAs has also assessed in PCa. For instance, expression levels of hsa_circ_0000437, hsa_circ_0000280, and circ_5017 have been correlated with poor survival of patients (97). Moreover, expression of circABCC4 has been associated with advanced clinicopathological features including higher tumor stage, metastasis and poor clinical outcomes (53). Over-expression of circMBOAT2 has also indicated shorted disease-free survival in two independent cohorts of PCa patients (30). Similarly, over-expression of circFOXO3 has been associated with advanced Gleason score and shorter overall survival of PCa patients (31).

Taken together, these results suggest circRNAs as ideal candidates to be used as biomarkers for diagnosis, prediction of prognosis and also provide therapeutical targets in treatment of PCa. Further studies are required to bring the circRNAs to clinical settings as useful tools with diagnostic, prognostic and therapeutical applications. **Table 3** shows the studies which have assessed the diagnostic, prognostic or clinical significance values of circRNAs in PCa.

## Discussion

CircRNAs are a novel type of ncRNAs for which some regulatory functions are known. Changes in their expression have been found in several disorders especially cancer. High-throughput technologies have helped identification of a vast number of circRNAs which exhibit dysregulation including down- or up-regulation in cancer tissues compared to NATs. Experimental and functional analyses have shown up-regulated circRNAs act as oncogenes, which promote tumorigenicity in cell studies. Conversely, down-regulated circRNAs play role as tumor suppressors and inhibit tumorigenic behaviors of cancer cell lines. In the majority of circRNAs, existence of interactions between them and miRNAs has revealed a mechanism through which circRNAs exert their roles and regulate cellular processes especially cell cycle. Precise understanding of action mechanisms may help finding therapeutic targets for cancer therapy. Clinical assessments, also have unveiled circRNAs as ideal candidates for diagnostic and prognostic applications. Similar to many cancers, a number of circRNAs have been identified to be dysregulated in PCa. In this review, we assessed the preeminent studies on the role of circRNAs in PCa in two categories of down- (**Table 1**) and up-regulated (**Table 2**) circRNAs focusing on functional experiments. Oncogenic circRNAs promote tumorigenicity *via* increasing cell proliferation, migration, and invasion *in vitro* and tumor growth and metastasis *in vivo*. Sponged miRNAs have been recognized in the majority of studies, through them circRNAs exert their roles *via* an axis which finally affects expression or activity of oncogenes or tumor suppressors, or directly influence the cell cycle.

miR-204-5p, miR-3160-5p, miR-548, miR-1182, miR-1271-5p, miR‐29a‐3p, miR-206, miR-185-3p, miR-29a, miR-193a-3p, miR-338-3p, miR-1299, miR-501-3p, miR-378a-3p, miR-124-3p, miR‐152‐3p, miR-647, miR-515-5p, miR-330-3p, miR-30b-5p, miR-615-5p, miR-326, miR-497, miR-7, miR-1238 and miR-646 are among the most important cancer-related miRNAs being sponged by circRNAs. The sponging effects of circRNAs on these miRNAs have a crucial role in the regulation of activity of cancer-related pathways. CircFOXO3/miR-29a‐3p, circ_0057558/miR-206, circ_0088233/miR-185-3p, circMYLK/miR-29a, circHIPK3/miR-193a-3p, circHIPK3/miR-338-3p, circHIPK3/miR-212, circ_0006404/miR-1299, circ-ZNF609/miR-501-3p, circPDHX/miR-378a-3p, circ-TRPS1/miR-124-3p, circNOLC1/miR-647 are examples of circRNAs/miRNAs axes with crucial roles in the pathogenesis of PCa.

Furthermore, diagnostic and prognostic values of circRNAs have been reviewed. Acceptable values have been reported for a set of circRNAs in PCa which suggest diagnostic and prognostic potentials of circRNAs. Some studies have proposed the role of circRNAs for easy, quick and less invasive diagnosis and prediction of prognosis of PCa patients based on their expression levels in liquid biopsies. However, based on the heterogeneous pattern of expression of circRNAs among patients, multi-gene panels are more promising than individual circRNAs. In addition, they may have therapeutic potentials, however further studies are required to utilize the potentials of circRNAs in clinical settings. It is also necessary to appraise expression of circRNAs in different settings to find possible factors that affect their expression in various cellular contexts.

## Author Contributions

SN, ArB, and SG-F wrote the draft and revised it. MT designed and supervised the study. AbB, BMH, and EJ collected the data and designed the figures and tables. All authors contributed to the article and approved the submitted version.

## Conflict of Interest

The authors declare that the research was conducted in the absence of any commercial or financial relationships that could be construed as a potential conflict of interest.

## Publisher’s Note

All claims expressed in this article are solely those of the authors and do not necessarily represent those of their affiliated organizations, or those of the publisher, the editors and the reviewers. Any product that may be evaluated in this article, or claim that may be made by its manufacturer, is not guaranteed or endorsed by the publisher.
